# Supplementary material for: Laser Irradiation Synthesis of AuPd Alloy with Decreased Alloying Degree for Efficient Ethanol Oxidation Reaction
Source: Materials (Basel). 2024 Apr 18;17(8):1876. doi: 10.3390/ma17081876 (PMC11052525; doi:10.3390/ma17081876)
Supplement: Supplementary file 1 [file materials-17-01876-s001.zip › materials-2952554-supplementary.pdf]

Supporting Information

Laser Irradiation Synthesis of AuPd Alloy with Decreased Alloying Degree for Efficient Ethanol Oxidation Reaction

Nan Jiang [1,2,3,†], Liye Zhu [1,2,3,†], Peng Liu [1], Pengju Zhang [1,2,3], Yuqi Gan [1,2,3], Yan Zhao [1,2,3,*] and Yijian Jiang [1,2,3]

[1] School of Physics and Optoelectronic Engineering, Beijing University of Technology, Beijing 100124, China; jiangnan@emails.bjut.edu.cn (N.J.); zly2019@emails.bjut.edu.cn (L.Z.); tianchenhe@emails.bjut.edu.cn (P.L.); z176816@emails.bjut.edu.cn (P.Z.); ganyq@emails.bjut.edu.cn (Y.G.); xishuaibin@emails.bjut.edu.cn (Y.J.)
[2] Key Laboratory of Trans-Scale Laser Manufacturing Technology, Beijing University of Technology, Ministry of Education, Beijing 100124, China
[3] Beijing Engineering Research Centre of Laser Technology, Beijing University of Technology, Beijing 100124, China
* Correspondence: zhaoyan@bjut.edu.cn
† These authors contributed equally to this work.

Table of Contents

S1. Materials and methods

S2. Supplementary Figures and Tables (Fig. S1-S7, Table S1-S7)

S1. Materials and methods

Materials

Ultrapure water (>18.2 MΩ) was used to prepare all the aqueous solutions. Aladdin Industrial Co., Ltd. provided L-ascorbic acid ($C_6H_8O_6$, AA, 99%), ethanol absolute (C_2H_6O, AR, 99.8%), potassium hydroxide (KOH, GR, 85.0%), and Borane-tert-Butylamine Complex (95%). Alfa Aesar Co., Ltd. supplied Nafion solution (5 wt.%) and sodium borohydride ($NaBH_4$, AR, 99%). Bide Pharmatech Co., Ltd. supplied 1,2,3,4-Tetrahydronaphthalene (99.81%). $HAuCl_4{\cdot}4H_2O$ was purchased from Tianjin Guangfu Fine Chemical Research Institute. Shanghai Macklin Biochemical Co., Ltd. provided palladium (II) chloride and carbon black. Premetek Co., Ltd. supplied commercial Pd/C (20% on Vulcan XC-72). Rhawn reagent Co., Ltd. provided OAm and EG. Beijing Yili Fine Chemicals Co., Ltd. supplied hydrochloric acid (GR). All reagents were of analytical grade and used as received without further purification.

Characterizations

Transmission electron microscopy (TEM) analysis was carried out using a FEI-Tecnai-Talos instrument at 300 kV. The FEI Titan-Themis 300, equipped with a probe Cs corrector, was employed for aberration-corrected high-angle annular dark-field scanning transmission electron microscopy (AC-HAADF-STEM) and associated energy-dispersive X-ray spectroscopy (HAADF-STEM-EDX) studies. Inductively coupled plasma-optical emission spectrometry (ICP-OES) experiments were performed using a PerkinElmer Optima 8300. X-ray photoelectron spectroscopy (XPS) was conducted with a Thermo Fisher ESCALAB 250Xi electron spectrometer utilizing Al Kα radiation, with all binding energies referenced to the C 1s peak at 284.3 eV. X-ray diffraction (XRD) patterns were obtained with a Bruker D8 Advance X-ray diffractometer using Cu Kα radiation (λ = 0.15418 nm), with the angle range from 10° to 90° (2θ) and detected by a LynxEye-one-dimensional detector. X-ray Absorption Fine Structure (XAFS) measurements were taken at the 1W2B beamline of the Beijing Electron-Positron Collider (BEPC) synchrotron radiation facility.

Electrochemical measurement

For the preparation of catalyst ink, the L-AuPd NPs/C was ultrasonically dispersed in 1 mL ethanol with 20 μL nafion solution (5wt%). 5 μL catalyst ink was casted onto the glassy carbon electrode and dried in air. All electrochemical measurements were conducted on an electrochemical workstation (CHI-760E) at room temperature. The catalyst-modified glassy carbon electrode (GCE, 5 mm diameter) was used as the working electrode, a saturated calomel electrode (SCE) and a Pt wire were used as the reference electrode and counter electrode, respectively. Cyclic voltammetry (CV) measurement (−1.0 ~ 0.2 V vs. SCE) was recorded in 1 M KOH after a steady curve was obtained. Ethanol oxidation reaction (EOR) performance was tested in 1 M KOH and 1 M CH_3CH_2OH solution with a sweep rate of 50 $mV{\cdot}s^{-1}$. Chronoamperometric (CA) measurements were performed at −0.3 V vs. SCE with a sweep rate of 50 $mV{\cdot}s^{-1}$. The potentials are converted to reversible hydrogen electrode (RHE) according to the Nernst equation:

$$E_{RHE} = E_{SCE} + 0.059 * pH + E_0 \text{ (}E_0\text{ equals to 0.241 V at 25 ºC)} \quad (S1)$$

CV measurements were conducted to determine the electrochemical specific surface area (ECSA):

$$ECSA = Q_{PdO/Pd} / (0.405 \text{ mC cm}^{-2} \times m_{Pd}) \quad (S2)$$

where $Q_{PdO/Pd}$ is the integral charge of the Pd oxides reduction, m_{Pd} is the mass loading of Pd on the glassy carbon electrode, and 0.405 mC cm^{-2} is a constant indicating the charge transfer for the formation of PdO monolayer on 1 cm^2 Pd metal surface.

DFT Calculation

DFT calculations were performed by Vienna ab initio simulation package (VASP) [52]. In the calculation, the electron wave function was expanded with the plane wave basis set. The projection augmented plane wave pseudopotential (PAW) was used to describe the interaction between the ion and the valence electron [53]. Generalized gradient approximation (GGA) of Perdew-Burke-Ernzerhof (PBE) approach was used to deal with the electron exchange correlation energy [54]. In all calculations, a cut-off energy of 450 eV was used for the plane wave basis set and a Monkhorst-Pack mesh of 3×3×1 was used

in K-sampling [55]. The convergence standard for total energy and force were set to 10^{-5} eV/atom and 0.03 eV/Å, respectively. Dispersion corrections were included via the zero-damping DFT-D3 correction method of Grimme in VASP [53]. The adsorption energy is calculated by the following:

$$E_{ads}=E_{total} - E_{slab} - E_{adsorbate} \quad \text{(S3)}$$

where E_{ads} is the adsorption energy, E_{total} is the total energy of the system with adsorbate, E_{slab} is the energy of the slab and $E_{adsorbate}$ is the energy of the isolated adsorbate in the gas phase. Vibrational frequencies of adsorbed intermediates and molecules in the gas phase were calculated with density-functional perturbation theory (DFPT). And all the thermodynamic energies were calculated at 298.15 K and 1 atm using VASPKIT package [56]. Gibbs free energies (G) of the adsorbed intermediates were calculated by：

$$G = H - TS = E_{DFT} + E_{ZPE} + E_{H} – TS \quad \text{(S4)}$$

where E_{DFT} is the total energy given by VASP calculations, E_{ZPE} is the zero-point energy correction, E_{H} is the enthalpy contribution and TS is entropy contribution. The ideal gas approximation and the harmonic approximation were used for molecules in gas phase and adsorbates, respectively. The free energy of one OH^- and H^+ was calculated by

$$G (OH^-) = G(H_2O(l)) - G(H^+) \text{ and } G (H^+) = 1/2G(H_2). \quad \text{(S5)}$$

For all slab models, a 20 Å vacuum layer was added to avoid the interaction between the mirrors. Before the geometrical optimization, the bottom two atomic layers were fixed and the other two layers were allowed to relax.

S2. Supplementary Figures and Tables

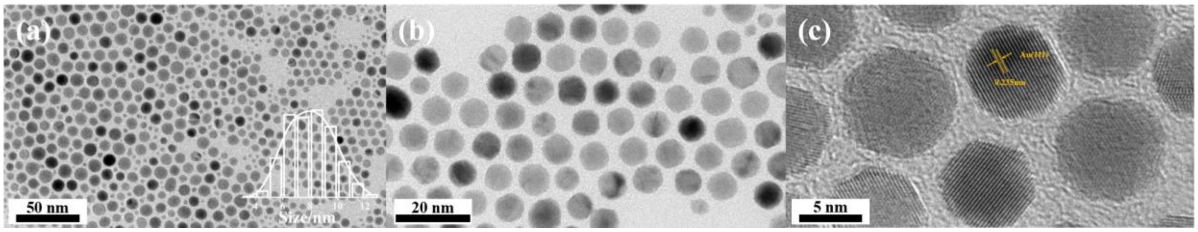

Figure S1. (**a**–**c**) TEM of chemically synthesized Au nanoparticles.

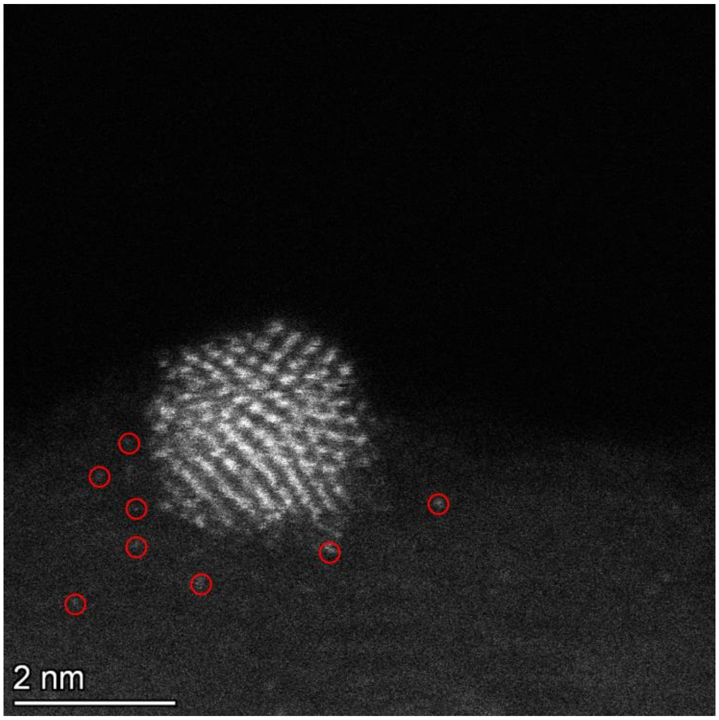

Figure S2. The Single atoms showed in red cycles ejected from the L-AuPd nanoparticles during the AC-HRTEM characterization.

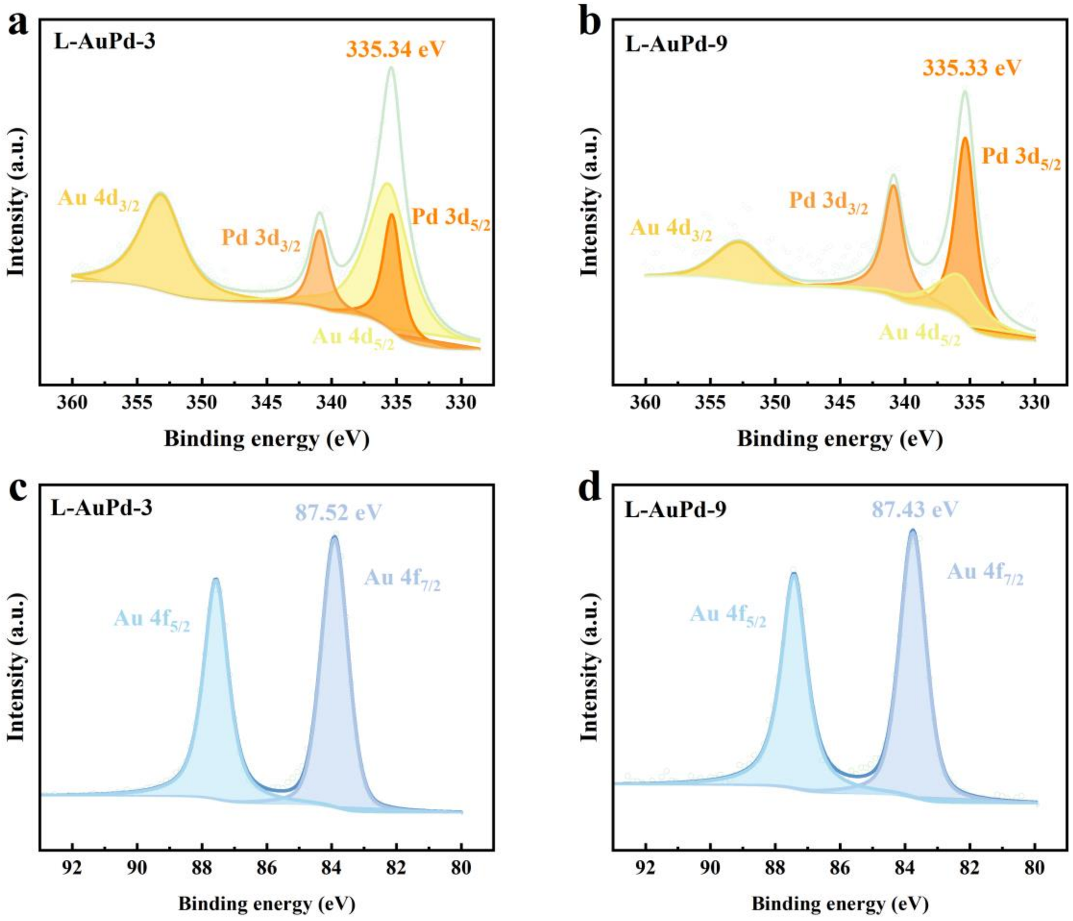

Figure S3. XPS spectra of Pd 3d signals of L-AuPd-3 (**a**) and L-AuPd-9 (**b**). XPS spectra of Au 4f signals of L-AuPd-3 (**c**) and L-AuPd-9 (**d**).

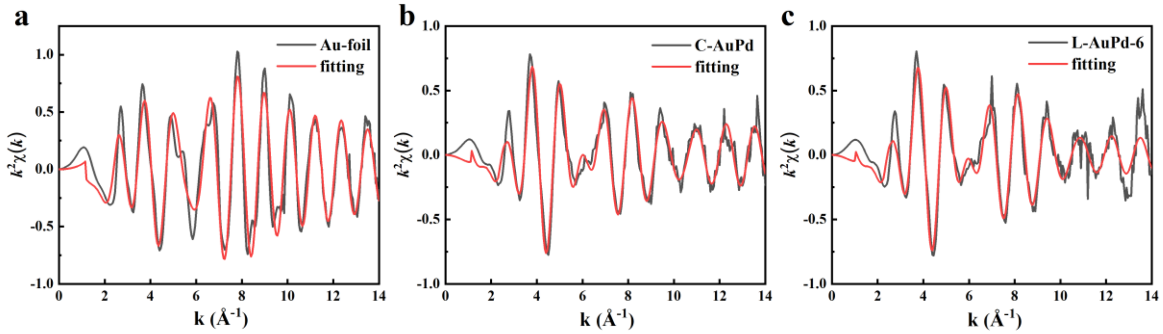

Figure S4. k^2-weighted EXAFS oscillations [$k^2\chi(k)$] of (**a**) Au foil, (**b**) C-AuPd and (**c**) L-AuPd-6.

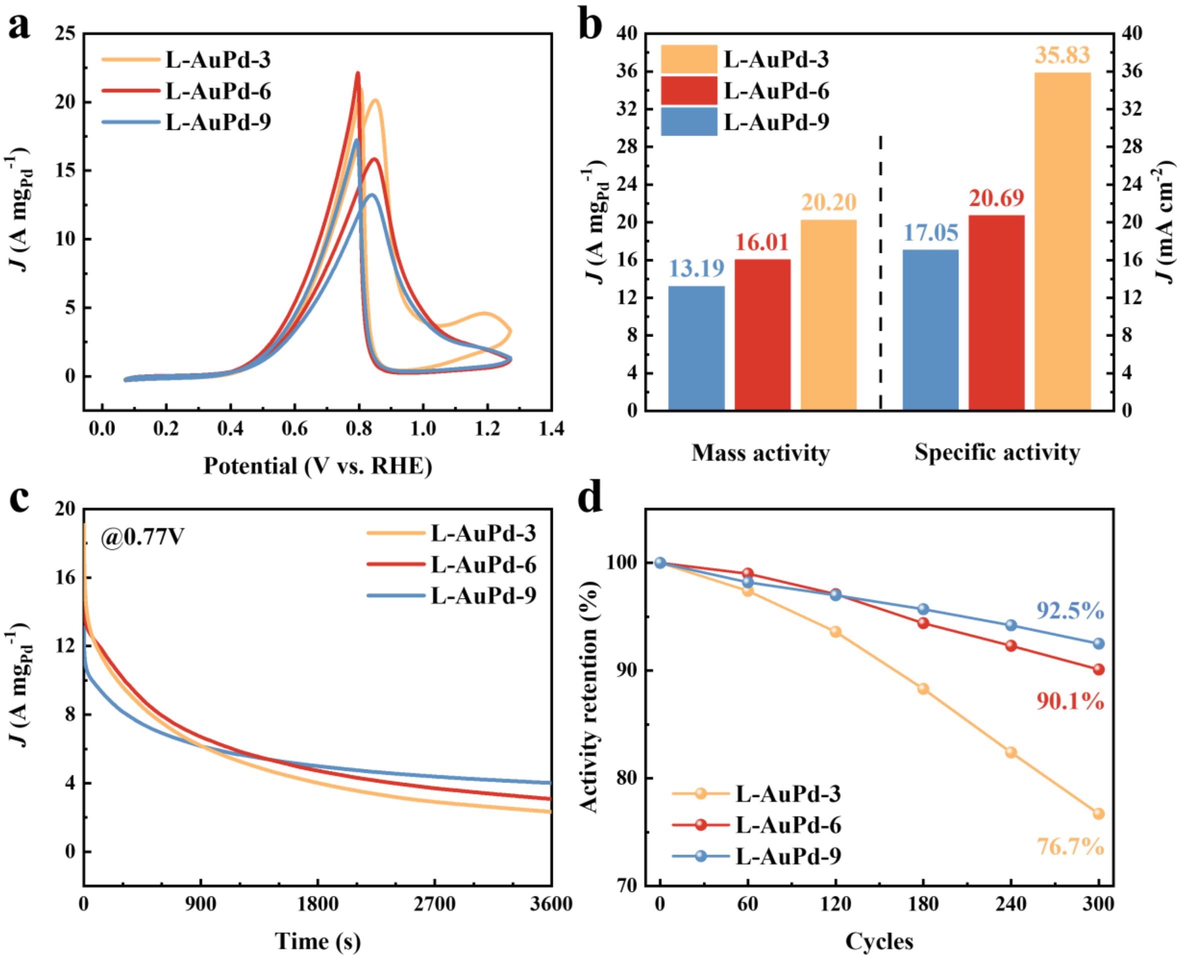

Figure S5. The performance for EOR of the L-AuPd nanoparticles synthesized with different irradiation time. (**a**) CV profiles. (**b**) Mass activities and specific activities. (**c**) CA curves. (**d**) ADT results.

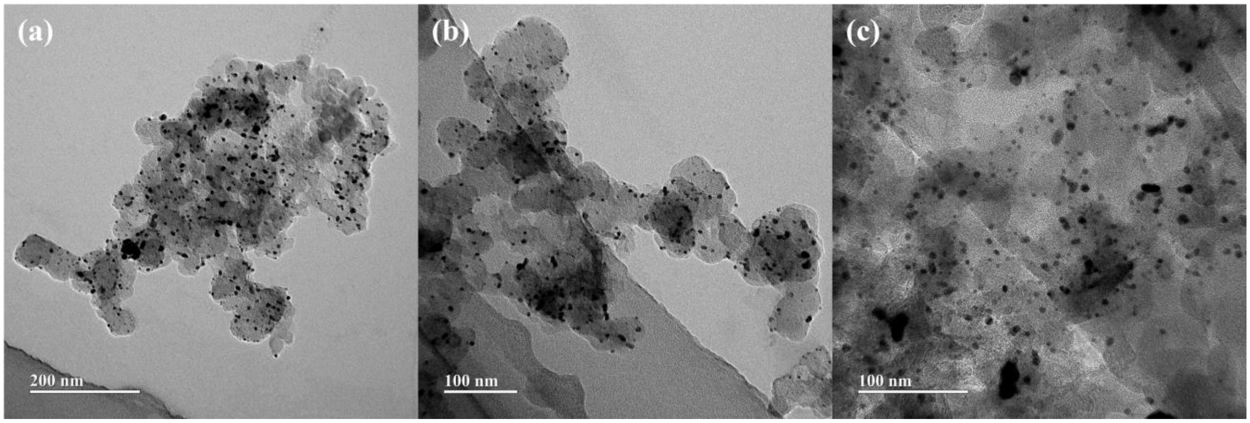

Figure S6. (**a**–**c**) TEM image of L-AuPd nanoparticles/C after ADT test.

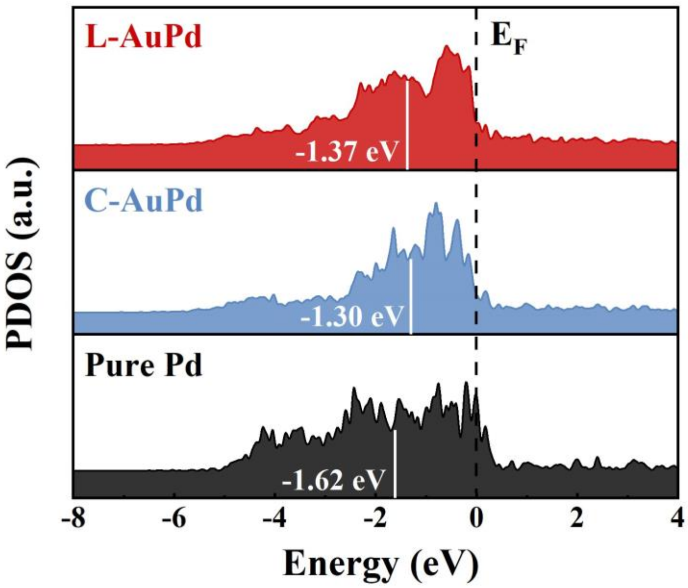

Figure S7. PDOS profile of surface Pd atom on L-AuPd, C-AuPd and pure Pd.

Table S1. ICP-OES results of L-AuPd nanoparticles and C-AuPd.

Samples	Au (At. %)	Pd (At. %)
L-AuPd-3	72.5	27.5
L-AuPd-6	50	50
L-AuPd-9	48.7	51.3
C-AuPd	50	50

Table S2. The difference (Δd) between d_{XRD} and d_{Vegard} of L-AuPd nanoparticles and C-AuPd. (Δd=d_{XRD}-d_{Vegard}).

Samples	d_{XRD} (nm)	d_{Vegard} (nm)	Δd (nm)
L-AuPd-3	0.2357	0.2343	0.00145
L-AuPd-6	0.2329	0.2300	0.00286
L-AuPd-9	0.2308	0.2295	0.00131
C-AuPd	0.2297	0.2300	-0.00034

Table S3. Summarized data of BEs of Pd 3d and Au 4f signals of L-AuPd nanoparticles and C-AuPd.

**Sample**		**Pd**			**Au**	
	Pd $3d_{5/2}$ Peak [eV]	Pd $3d_{3/2}$ Peak [eV]	ΔPd $3d_{5/2}$ [eV]	Au $4f_{7/2}$ Peak [eV]	Au $4f_{5/2}$ Peak [eV]	ΔAu $4f_{7/2}$ [eV]
Pd/C	335.10	340.36	0	-	-	-
Au/C	-	-	-	83.91	87.58	0
L-AuPd-3	335.34	340.60	+0.24	83.85	87.52	-0.06
L-AuPd-6	335.40	340.66	+0.30	83.75	87.42	-0.16
L-AuPd-9	335.33	340.59	+0.23	83.76	87.43	-0.15
C-AuPd	335.04	340.30	-0.06	83.61	87.28	-0.30

Table S4. EXAFS fitting parameters at the Au L_3-edge for various samples (S_0^2=0.82 from Au-

foil).

	shell	CN[a]	R[b](Å)	σ[2c](Å[2])	ΔE$_0$[d](eV)	R factor
Au-foil	Au-Au	12	2.86±0.01	0.0077	5.3±0.3	0.0049
C-AuPd	Au-Pd	4.1±0.1	2.79±0.01	0.0063	4.9±0.4	0.0046
	Au-Au	7.1±0.2	2.82±0.01	0.0076		
L-AuPd-6	Au-Pd	3.7±0.1	2.79±0.01	0.0066	4.0±0.4	0.0042
	Au-Au	7.7±0.3	2.81±0.01	0.0089		

[a]*CN*: coordination numbers; [b]*R*: bond distance; [c]σ^2: Debye-Waller factors; [d] ΔE_0: the inner potential correction. R factor: goodness of fit. Error bounds that characterize the structural parameters obtained by EXAFS spectroscopy were estimated as CN±20%; R ± 1%; σ^2 ± 20%.

Table S5. EOR activities of L-AuPd nanoparticles catalysts and Pd-based catalysts already reported in the literature.

Catalyst	Synthesis method	Electrolyte	MA (A mg_{Pd}^{-1})	SA (mA cm^{-2})	Ref
L-AuPd-3	Laser irradiation in liquids	1 M KOH + 1 M ethanol	20.20	35.83	This work
L-AuPd-6	Laser irradiation in liquids	1 M KOH + 1 M ethanol	16.01	20.69	This work
L-AuPd-9	Laser irradiation in liquids	1 M KOH + 1 M ethanol	13.19	17.05	This work
Au-Pd-1	Sol-gel	1 M KOH + 1 M ethanol	12.99	NA	[57]
Ag@AgPd	Hydrothermal	1 M KOH + 1 M ethanol	12.70	18.20	[58]
Au_8Pd_3 alloy nanowires	Chemical reduction	1 M KOH + 1 M ethanol	10.57	6.20	[59]
AuPd aerogel	Laser ablation in liquids and Laser irradiation in liquids	1 M KOH + 1 M ethanol	9.75	NA	[60]
Porous $Pd_{97}W_3$	Solvothermal	1 M KOH + 1 M ethanol	9.50	9.10	[61]
Au-Pd aerogel	Freeze-thaw	1 M KOH + 1 M ethanol	8.40	NA	[62]
Se-Pd NSs/C	Solvothermal	1 M KOH + 1 M ethanol	6.94	8.78	[63]
Fcc-2H-fcc Au@Pd	Solvothermal	1 M KOH + 1 M ethanol	6.82	13.77	[64]
$L1_2$ PdCuSn	Solvothermal	1 M KOH + 1 M ethanol	6.22	NA	[65]
PdBi nanorings	Solvothermal	1 M KOH + 1 M ethanol	5.78	11.93	[66]
Porous PdPtNi nanosheets	Solvothermal	1 M KOH + 1 M ethanol	5.12	4.16	[67]
PdAu nanospheres	Hydrothermal	1 M KOH + 1 M ethanol	5.11	10.30	[68]
PdMoH bimetallene	Solvothermal	1 M KOH + 1 M ethanol	3.56	6.06	[69]
Pd/NCB@NGS-2	Hydrothermal	1 M KOH + 1 M ethanol	2.70	5.23	[70]
Pd@Ni nanoparticles	Chemical reduction	1 M KOH + 1 M ethanol	1.09	NA	[71]

Table S6. ECSA data of L-AuPd NPs, C-AuPd and Pd/C catalysts.

**Catalyst**	**ECSA ($m^2\ g_{Pd}^{-1}$)**
L-AuPd-3	56.38
L-AuPd-6	50.20
L-AuPd-9	47.75
C-AuPd	29.54
Pd/C	28.66

Table S7. The reaction pathway for EOR is list as following.

State	Reaction pathway
1	$CH_3CH_2OH + * \rightarrow CH_3CH_2OH*$
2	$CH_3CH_2OH* + OH^- \rightarrow CH_3CHOH* + H_2O + e^-$
3	$CH_3CHOH* + OH^- \rightarrow CH_3CHO* + H_2O + e^-$
4	$CH_3CHO* + OH^- \rightarrow CH_3CO* + H_2O + e^-$
5	$CH_3CO* + OH^- \rightarrow CH_3COOH* + e^-$
6	$CH_3COOH* \rightarrow CH_3COOH + *$
